# Exploration of Entrepreneurship Education by Linear Regression and Psychological Factor Analysis

**DOI:** 10.3389/fpsyg.2020.02045

**Published:** 2020-08-12

**Authors:** Ke Mu, Qin Shi, Yonghong Ma, Jiao Tan

**Affiliations:** ^1^School of Health Management, Xi’an Medical University, Xi’an, China; ^2^School of Public Health, Xi’an Medical University, Xi’an, China; ^3^Department of Epidemiology and Health Statistics, School of Public Health, Xi’an Medical University, Xi’an, China

**Keywords:** entrepreneurial performance, entrepreneurial self-efficacy, psychological factors, entrepreneurial innovation learning, school organizational support, social interaction, linear regression analysis

## Abstract

To study the improvement of the entrepreneurial performance of start-ups and achieve an organic combination of entrepreneurship education and entrepreneurial performance, the entrepreneurial group of college students was taken as the springboard to discuss the entrepreneurial performance of start-ups initiated by college students. First, through questionnaire design and scale selection, the results of the questionnaire survey and the reliability and validity of the scale tool were tested. Second, the variable of college students’ entrepreneurial self-efficacy based on psychological factor analysis was introduced. The correlations among entrepreneurship education, entrepreneurial self-efficacy, and entrepreneurial performance were analyzed. Finally, the intermediary role of entrepreneurial self-efficacy was verified by using the hierarchical linear regression analysis method combined with the BARON three-part verification method. The results show that the response rate of the questionnaire is 92%, and the selection of research samples is scientific. The Cronbach’s α reliability coefficients corresponding to each scale are all above 0.8, and the common factor variances are all above 0.7. Therefore, the reliability and validity of scale tools are good. Entrepreneurship education, entrepreneurial self-efficacy, and entrepreneurial performance are significantly correlated with each other. The college students’ entrepreneurial self-efficacy has a good explanatory ability and plays an intermediary role; in the entrepreneurial innovation learning dimension, its corresponding β = 0.257; in the dimension of school organizational support, the corresponding β = 0.439; in the dimension of social interaction, the corresponding β = 0.238. The results have a reference value for studying entrepreneurship education and the improvement of entrepreneurial performance from a psychological level.

## Introduction

Currently, with the rapid development of information technology, many opportunities for innovation and entrepreneurship have emerged, and entrepreneurship education has attracted attention (Kalafatis and Lemos, 2017). Entrepreneurship education refers to a teaching process, which is embodied in entrepreneurship or enterprise development and progress. For entrepreneurship education, scholars hold different views, but the two representative views are as follows. One is that entrepreneurship education is a combination of learning opportunities and education models, which is beneficial to the improvement of participants’ social practical ability (Oliveira et al., 2019). Another view is that entrepreneurship education is an important form of quality education (Štrukelj et al., 2019). Under the background of rapid development, such as computer information technology and big data analysis, there is an urgent need for more and better innovative and entrepreneurial talents. Whether it is an individual or an organization, the innovation and creative ability and level are inseparable from the development of the social economy (Aykan et al., 2019). From the perspective of measuring entrepreneurship education, entrepreneurial performance is one of the key impact indicators. This evaluation indicator can provide a more detailed evaluation of entrepreneurial innovation capabilities and the effectiveness of entrepreneurship education (Ameer and Khan, 2020). In the evaluation and selection of innovation and entrepreneurship, the psychological factors of individual participants will inevitably have an impact on the entrepreneurial process and results. However, the research on the psychological factors on entrepreneurial performance and entrepreneurship education is still rare.

Therefore, college entrepreneurs were taken as research samples, who came from different types of colleges and universities in Xi’an City, Shaanxi Province. The entrepreneurial self-efficacy of college students was introduced as an intermediary variable based on the analysis of psychological factors. The linear regression analysis method was utilized to analyze the correlations among entrepreneurship education in colleges, entrepreneurial performance, and self-efficacy (Liu et al., 2019). Also, the intermediary role of entrepreneurial self-efficacy was verified through data quantification. It is hoped to provide some data references for the improvement of the entrepreneurial performance of start-ups in the context of entrepreneurship education (Chen et al., 2019).

## Literature Review

### International Research Progress

Globally, there have been many studies on entrepreneurship education. Tingey et al. (2020) explored the impact of the ABG entrepreneurial education program on entrepreneurial knowledge, economic empowerment, and social welfare; they found that entrepreneurial education intervention has an important role. By taking the entrepreneurial education assessment theory as the foundation, Shekhar et al. (2018) provided a basic framework for the development of entrepreneurship projects for college students through the establishment of the conceptual model of student entrepreneurship education. Sher et al. (2017) surveyed the entrepreneurial willingness of Pakistani agriculture-related graduates, and the results showed that the guidance of successful entrepreneurs and experts was very helpful in promoting student entrepreneurship activities. Leong et al. (2017) studied the development path of entrepreneurship and innovation education in medical specialties, and the results showed that strengthening the innovation and integration of related courses could provide great help for the development of students. Asimakopoulos et al. (2019) explored the relationship between entrepreneurship education, entrepreneurial intention, and entrepreneurial self-efficacy using engineering students as the research samples. The results suggested that entrepreneurial education had a positive regulatory effect on the relationship between entrepreneurial self-efficacy and entrepreneurial intention; at the same time, it also had a positive correlation with the intention of entrepreneurial activities. Basil and Karatuna (2017) studied the entrepreneurial self-efficacy of college students in Poland and Turkey.

### Research Progress in China

In China, there are a variety of studies on entrepreneurship education. Cai and Kong (2017) took the students of Xi’an Medical University as research objects, analyzed and discussed the relationship between entrepreneurship education and entrepreneurial intention, and found that entrepreneurial college education had a significant impact on college students’ entrepreneurial intention. Liang et al. (2018) believed that entrepreneurs with entrepreneurial enthusiasm and passion had an important impact on the long-term development of enterprises. Wang et al. (2019) analyzed and explored the creativity and innovative spirit of doctors; the results showed that doctors at different age levels and different educational levels have differences in innovation and creativity.

As shown in the previous researches, there have been many studies on the relationship between entrepreneurial education and entrepreneurial self-efficacy. However, few studies were conducted in combination with several aspects, and the researches that consider the psychological factors of individual participants are even more lacking (Obschonka et al., 2018). On this basis, the psychological elements were introduced into the exploration and research of entrepreneurship education, and the relationship between entrepreneurial performance and entrepreneurship education was analyzed and discussed.

## Materials and Methods

### Entrepreneurial Performance

Generally, performance is characterized by objective evaluation and consideration of organizational or individual behaviors. The definition of entrepreneurial performance is mainly rendered from three different aspects: result-oriented, behavioral process-oriented, and comprehensive impact (Giudice et al., 2017; Li et al., 2019). Many theories have been built for the exploration of entrepreneurial performance. Among them, there were environmental theory, strategic adaptation theory, and resource theory occupy important positions. Environments such as politics and economy are all part of the entrepreneurial environment; theories such as basic entrepreneurial theory and efficiency theory are all part of entrepreneurial resources. Considering different dimensions and different indicators, to analyze the factors of entrepreneurial performance, empirical analysis and testing are common methods. Currently, scholars have found that organizational goals, core technologies, and team building are essential in improving entrepreneurial performance. The individual characteristics of entrepreneurs and the entrepreneurial environment are also vital in entrepreneurial performance. Some studies have attributed the individual psychological factors of entrepreneurs as the subjective reasons that affect entrepreneurial performance; they have also attributed the entrepreneurial environment and entrepreneurial resources to objective reasons that affect entrepreneurial performance (Hormiga et al., 2017). From the perspective of evaluation indicators, the financial indicators, development prospects, and the creation of innovation levels have been introduced into the measurement indicators. In general, the current research on entrepreneurship performance improvement has less integration of multiple dimensions (Rogoza et al., 2018; Yu et al., 2020). Also, there is currently less research on entrepreneurship education and entrepreneurial performance of college students.

### Entrepreneurial Self-Efficacy

In the process of entrepreneurship, the reality represented by entrepreneurial self-efficacy is a psychological feeling of entrepreneurs, which can be attributed to the level of psychological elements. Specifically, it is a manifestation of confidence in entrepreneurial results and is a positive component that can promote successful entrepreneurship (Qu et al., 2017; Borrero, 2018). Therefore, it is believed that entrepreneurial self-efficacy will also have an impact on entrepreneurial performance, which is introduced as an intermediary variable that affects entrepreneurial performance. In the evaluation of entrepreneurial self-efficacy, the division of dimensions is the major method. Among them, the exploratory analysis and the New General Self-Efficacy Scale are two major tools for entrepreneurial self-efficacy measurement.

### Psychological Factors, Entrepreneurial Self-Efficacy, and Entrepreneurial Performance

**FIGURE 1:**
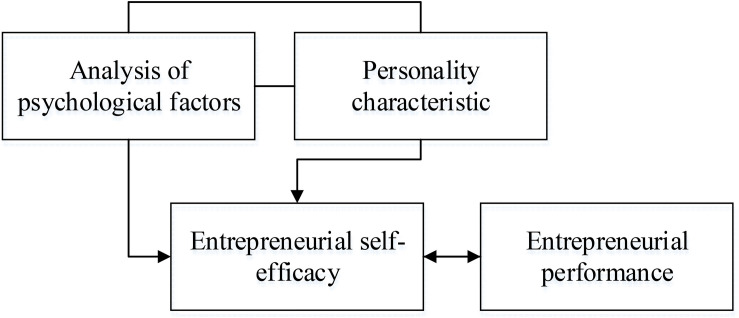
Correlations among psychological factors, entrepreneurial self-efficacy, and entrepreneurial performance.

There is a significant difference in the level of individual creativity. As one of the important abilities of individuals, entrepreneurial self-efficacy is an important personality factor in terms of creativity. Previous research results have found that individuals with good entrepreneurial self-efficacy have more positive evaluations for themselves (Qian et al., 2018; Bryce et al., 2019). Therefore, they can participate more in school and improve their level of creativity. Entrepreneurial self-efficacy has a positive predictive effect on creativity. Therefore, it is very necessary to explore the role of entrepreneurial self-efficacy in creativity. From an individual perspective, it can also be considered that entrepreneurial self-efficacy is a psychological factor possessed by an individual, which belongs to the category of personality traits. Research has found that personality traits are significantly correlated to the physical and mental health of individuals, which also have predictive capabilities for individual physical and mental health. Personality traits have a significant correlation with job performance, and employees with internal control personality have better job performances than those with external control personality. Hence, in addition to personality traits, including self-efficacy, psychological elements are closely correlated to entrepreneurial performance. The correlations among psychological factors, entrepreneurial self-efficacy, and entrepreneurial performance are shown in Figure 1.

### Design of Research

#### Model Construction and Hypotheses

**FIGURE 2:**
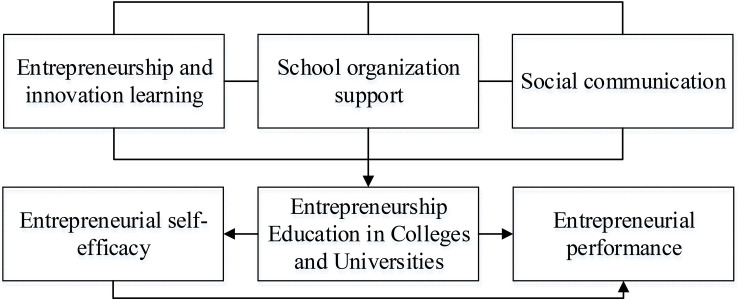
Model construction.

During the implementation of entrepreneurship education in colleges and universities, the inspection and summary feedback on the achievements of entrepreneurship education activities were ultimately realized in the form of entrepreneurial performance. At the same time, the establishment of the psychological element of individual entrepreneurial self-efficacy of college entrepreneurs had a huge influence on the effect of entrepreneurship. Therefore, the higher the entrepreneurial self-efficacy of individual entrepreneurs, the faster they can adapt to the competitive market environment. Furthermore, the psychological element of entrepreneurial self-efficacy is not a constant personality trait, which is constantly improved with learning and time. The development of entrepreneurship education in colleges and universities helps students promote the implementation of entrepreneurial practice, master the entrepreneurial skills that adapt to society, and embody a sense of entrepreneurial self-efficacy. Research has shown that college students who choose entrepreneurship-related courses show a higher level of entrepreneurial self-efficacy (Mollica et al., 2017). It is not difficult to find that the promotion of entrepreneurial events by college entrepreneurship education does not happen overnight. It must be achieved through psychological activities, which demonstrates the necessity of the psychological element of entrepreneurial self-efficacy as a mediator. As an important form of personality traits, entrepreneurial self-efficacy is an important psychological factor that affects entrepreneurship education. It has a positive effect on the realization of entrepreneurship and the improvement of entrepreneurial performance. Therefore, the introduction of the concept of entrepreneurial self-efficacy based on psychological factors is of great significance for the analysis of the correlation between entrepreneurship education and entrepreneurial performance, as well as the improvement of entrepreneurial performance. This also corresponds to the research hypotheses proposed later. Thus, the model established is shown in Figure 2, and the research hypotheses are proposed, as shown in Table 1.

**TABLE 1:** Proposal of research hypotheses.

**Hypothesis number**	**Concrete content**
H1	Entrepreneurial and innovation learning has a positive impact on college students’ entrepreneurial self-efficacy under the analysis of psychological factors.
H2	School organizational support has a positive impact on college students’ entrepreneurial self-efficacy under the analysis of psychological factors.
H3	Social interaction has a positive impact on college students’ entrepreneurial self-efficacy under the analysis of psychological factors.
H4	The self-efficacy of college students’ entrepreneurship under the analysis of psychological factors has a positive impact on entrepreneurial performance.
H5	Entrepreneurial and innovation learning has a positive impact on college students’ entrepreneurial self-efficacy under the analysis of psychological factors.
H6	School organizational support has a positive impact on college students’ entrepreneurial self-efficacy under the analysis of psychological factors.
H7	Social interaction has a positive impact on college students’ entrepreneurial self-efficacy under the analysis of psychological factors.
H8	The self-efficacy of college students’ entrepreneurship under the analysis of psychological factors plays an intermediary role in entrepreneurship education and entrepreneurship performance.

#### Questionnaire Design and Research Sample Selection

Considering that Xi’an has many colleges and universities and tremendous students, to ensure that the data are uniform, Shaanxi Normal University and Northwestern Polytechnical University in Xi’an were chosen as samples, and student entrepreneurs in these two universities were surveyed. According to differences in sex, educational levels, and other aspects, surveys were performed. For the design of the questionnaire, the suggestions of teachers and students were considered, and its content had been thoroughly considered. For the selection of individual research samples, there were two main focuses. One was the independent operation by student entrepreneurs, and the other was that the start-ups were the first company that students initiated. Considering that the development of entrepreneurship education was mainly carried out in colleges and universities, its main audience was college students; thus, participants of this questionnaire survey were college students. The questionnaire was filled in by a combination of an offline paper questionnaire and an online network questionnaire. The paper copies were distributed to selected universities. The invalid questionnaires, such as questionnaires with many blanks or highly blank options, were eliminated. To ensure the quality of the data collected by the questionnaire survey, before the questionnaire was issued, expert evaluation and internal testing were performed in advance (Kulczycki and Rozkosz, 2017), for example, if the results of the questionnaire options were highly consistent, and the correlation of the degree of discrimination could not be reflected in the respondents; this question would be removed. Afterward, the final version of the questionnaire was determined.

In the selection of measurement scales for each component research variable, the entrepreneurship education scale was based on the scale of student participation in innovation and entrepreneurship education, which contained three subscales for a total of 10 questions. For entrepreneurial self-efficacy based on psychological elements, the scale was based on the general self-efficacy scale, including five questions (Nepal et al., 2018). For entrepreneurial performance, the scale was based on the entrepreneurial performance scale, including a total of five questions (Zhang and Li, 2018). For the correlation measurement scale, the Likert scale was utilized (Hizo-Abes et al., 2018). The reliability and validity indicators were utilized to test the reliability of the scale. Among them, the reliability test was evaluated by the size of Cronbach’s α coefficient. For the validity test, the factor analysis method was combined with Kaiser–Meyer–Olkin (KMO) evaluation and Bartlett test. The specific evaluation indicators were the following: (1) the corresponding value of KMO was above 0.6, and (2) the statistical value of Bartlett test sig. was below 0.001. At this time, factor test analysis could be performed. The SPSS 26.0 software was used for statistical analysis. The composition of the measurement scale for each research variable is shown in Table 2.

**TABLE 2:** Composition of measurement scale for each research variable.

**Research variable dimension**		**Measurement problems**
Entrepreneurship Education	Entrepreneurship and innovation learning	1.1 Spend a lot of time on professional study.
		1.2 Participate in Entrepreneurship and innovation activities.
	School organizational support	2.1 Release of policy information on entrepreneurship and innovation.
		2.2 Opening of elective courses of entrepreneurship and innovation.
		2.3 Provide funds and venues for entrepreneurship and innovation activities.
		2.4 Entrepreneurship and innovation skills training.
	Social interaction	3.1 Resource integration and judgment ability can be improved.
		3.2 Improvement of project performance level.
		3.3 Explore entrepreneurial ideas.
		3.4 Participation in Entrepreneurship and innovation practice projects.
Entrepreneurial self-efficacy		4.1 Go all out to deal with the problem.
		4.2 It is easy in any situation.
		4.3 As long as we are diligent and practical, we can solve many problems.
		4.4 Believe in my social skills to cope with any situation.
		4.5 Be able to consider and solve problems from multiple perspectives.
Entrepreneurial performance		5.1 There are a few financial difficulties.
		5.2 The company is doing well.
		5.3 The company has existed for a long time.
		5.4 The growth rate of return on investment is higher than that of peers.
		5.5 The profit is considerable.

### Linear Regression Analysis

Linear regression analysis belongs to the quantitative analysis methods, which is widely used in the statistical analysis of data. The application of this method can determine the correlation between two or more variables (Khaled et al., 2019). Among them, beta is called the regression coefficient, also expressed as β. The standardized regression coefficient can characterize the correlation between the variables. The F statistic is the variance test for the entire regression model, and the corresponding significance level p is used to judge whether the result of the F F-test is significant. The determination coefficient R square (R^2^) and the adjusted R square (Adj.R^2^) characterize the fitting effect presented by the regression model, of which characterization results of Adj.R^2^ are more accurate. Therefore, these statistics in the linear regression analysis were selected to analyze and test the correlations among entrepreneurship education in colleges and universities, entrepreneurial self-efficacy, and the entrepreneurial performance based on psychological element analysis.

Given that entrepreneurship education in colleges and universities is a relatively broad concept, it is divided into three dimensions in the subsequent correlation research – —the entrepreneurship innovation learning (A1), the school organizational support (A2), and the social interaction (A3). According to theoretical analysis, it is believed that the personality traits of college students’ entrepreneurial self-efficacy, a psychological element, can be used as an intermediary variable in entrepreneurship education and entrepreneurial performance. To test the intermediary effect, the hierarchical linear regression analysis method was combined with the BARON duality innovation intermediary three-part verification method. The specific implementation process of the three-part verification method was to study first the independent variables and dependent variables. Then, it verified the entrepreneurial self-efficacy through the independent variables. Finally, through the hierarchical regression analysis method, these two steps were verified. If the regression coefficients of the corresponding independent variables and intermediary variables are significant, and the reduction of the significant coefficient occurs in the independent variables, the corresponding intermediary variables will have a partial intermediary role in them. Therefore, the third step is verified by using the college entrepreneurship education and entrepreneurial self-efficacy to explain entrepreneurial performance at the same time, thereby making judgments based on data changes in control variables.

## Results

### Analysis of Questionnaire Results

The demographic characteristics of the selected research samples are shown in Table 3.

**TABLE 3:** Demographic characteristics of the research samples.

**Influence parameter**	**Classification and composition**	**Percentage**
Sex	Male	57.5%
	Female	42.5%
Age	Over 27 years old	45%
	21–27 years old	48%
	20 and under years old	7%
Educational background	Master	21.9%
	Undergraduate	64.1%
	Junior college	14%
Years of establishment	More than 5 years	11.9%
	3–5 years	37.2%
	1–3 years	42.8%
	Within 1 year	8.1%
Industry	Traditional manufacturing	15%
	Science and technology service industry	58%
	Hi-tech industry	20%
	General service industry	7%

A total of 400 questionnaires were issued, and 380 questionnaires were recovered. After invalid questionnaires were removed, the total number of valid questionnaires was 350. The response rate of this questionnaire survey is 95%. The valid rate is 92%. As shown in the Table 3, the selected samples cover different sexes, different educational levels, and different industries, thereby laying a good foundation for follow-up exploration and analysis.

### Reliability and Validity Test of Selected Scales

For the entrepreneurship education scale, the entrepreneurial self-efficacy scale under the psychological elements, as well as the reliability test results of the entrepreneurial performance scale, are shown in Table 4.

**TABLE 4:** Results of reliability test.

**Research variables**	**Items**	**Cronbach’s α**
Entrepreneurship Education	1.1	0.916
	1.2	0.912
	2.1	0.905
	2.2	0.907
	2.3	0.905
	2.4	0.907
	3.1	0.905
	3.2	0.906
	3.3	0.912
	3.4	0.911
	The total scale	0.928
Entrepreneurial self-efficacy	4.1	0.899
	4.2	0.894
	4.3	0.882
	4.4	0.892
	4.5	0.896
	The total scale	0.918
Entrepreneurial performance	5.1	0.913
	5.2	0.904
	5.3	0.893
	5.4	0.911
	5.5	0.910
	The total scale	0.925

An analysis of the Cronbach’s α coefficient values in the figure shows that at the level of entrepreneurship education, the Cronbach’s α value corresponding to each question in the scale is above 0.9, and the comprehensive measurement value of the scale is 0.928. At the entrepreneurial self-efficacy level of the entrepreneurs’ psychological elements, the Cronbach’s α value corresponding to each question in the scale is above 0.8, and the comprehensive measurement value of the scale is 0.918. At the level of entrepreneurial performance, the Cronbach’s α value corresponding to each question in the scale is above 0.8, and the comprehensive measurement value of the scale is 0.925.

**FIGURE 3:**
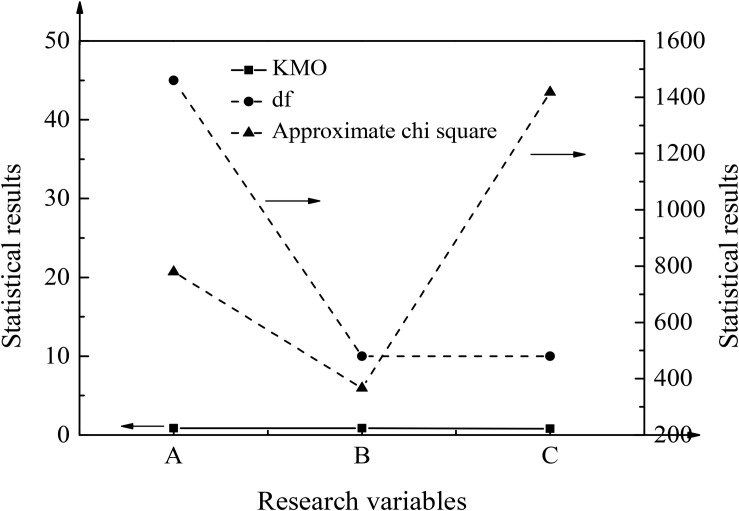
KMO and Bartlett test results. In the figure, the abscissa **(A)** represents entrepreneurship education, **(B)** represents entrepreneurial self-efficacy, and **(C)** represents entrepreneurial performance.

**FIGURE 4:**
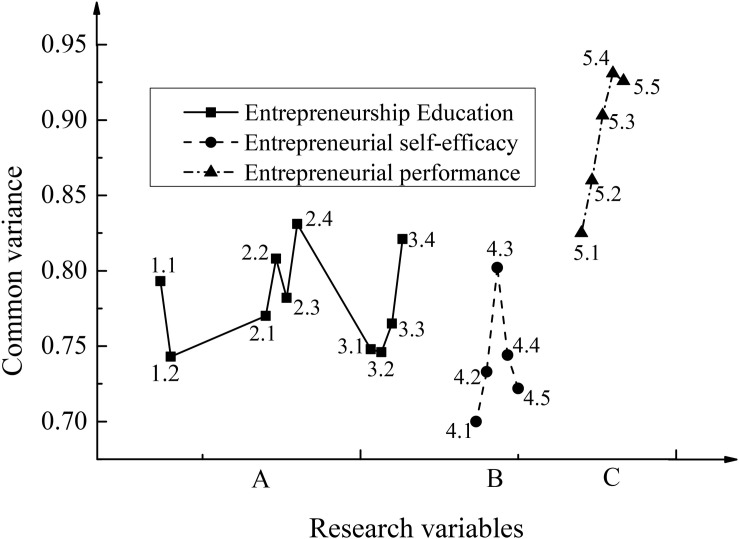
Results of common factor variance test for each scale. In the figure, 1.1–5.5 correspond to the questions in the scale.

For the entrepreneurship education scale, the entrepreneurial self-efficacy scale under the psychological factors, the KMO and Bartlett test results of the entrepreneurial performance scale, and the results of common factor variance test for each scale are shown in Figures 3, 4.

According to Figures 3, 4, the KMO value corresponding to entrepreneurship education is 0.834, which is above 0.8. The sig. value of Bartlett’s statistical value is 0.000, and the variance of the common factor corresponding to each question in the scale is above 0.7. The KMO value corresponding to the entrepreneurial self-efficacy is 0.866, which is above 0.8. The sig. value of Bartlett’s statistical value is 0.000, and the variance of the common factor corresponding to each question in the scale is also above 07. The KMO value corresponding to entrepreneurial performance is 0.813, which is above 0.8. The sig. = 0.000; the variance of the common factor of each question in the scale is still above 0.7.

### Linear Regression Analysis of Research Variables

**FIGURE 5:**
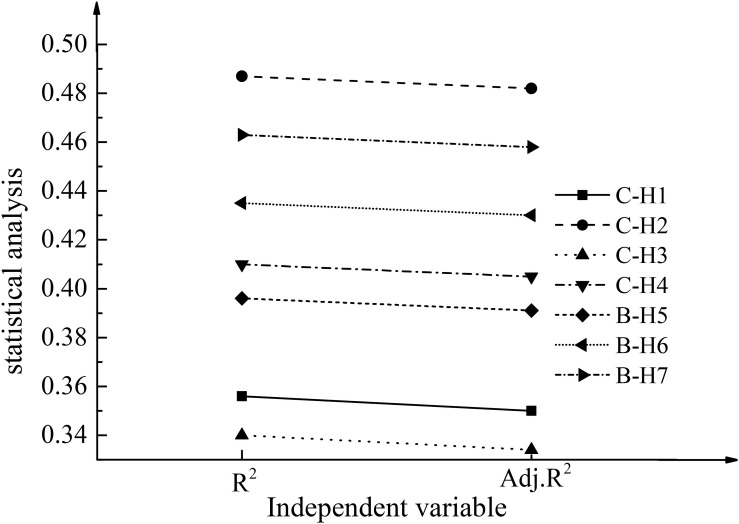
Results of linear regression analysis. C-H1 in the figure represents the R^2^ and Adj.R^2^ values corresponding to entrepreneurial performance under H1; the rest may be deduced by analogy.

For entrepreneurship education and entrepreneurial performance, the linear regression analysis results of the correlation between entrepreneurial self-efficacy and entrepreneurial performance based on psychological elements are shown in Table 5 and Figure 5.

**TABLE 5:** Results of linear regression analysis.

**Independent variable**	**H1**	**H2**	**H3**	**H4**	**H5**	**H6**	**H7**
	C	C	C	C	B	B	B
A1	0.522***				0.551**		
A2		0.665**				0.623**	
A3			0.577**				0.665**
B				0.676***			

*^∗∗^p < 0.01; ^∗∗∗^p < 0.001.*

The analysis of Table 5 shows that the three components of entrepreneurship education in colleges and universities, i.e., entrepreneurial innovation learning, school organizational support, and social interaction activities, have a significant linear correlation with the entrepreneurial performance of college students. Among them, a significant positive correlation exists between entrepreneurial innovation learning activities and college students’ entrepreneurial performance, with specific performance of 0.522 and a significant level of *p* < 0.001. Accordingly, H1 is reasonable, and it can be verified. Also, school organizational support and social interaction have a positive effect on college students’ entrepreneurial performance. The specific performance of the former is 0.665, with a significance level of *p* < 0.01; the specific performance of the latter is 0.577, with a significance level of *p* < 0.01. Therefore, H2 and H3 are reasonable.

In terms of the correlation between entrepreneurial self-efficacy and entrepreneurial performance based on the psychological elements, the corresponding *R*^2^ = 0.411 and Adj.R^2^ = 0.406, in which there is a significant positive correlation between the entrepreneurship innovation learning dimension and entrepreneurial performance. The correlation is specifically expressed as β = 0.676, *p* < 0.001. Therefore, they have a significant linear correlation. Hence, the H4 is reasonable. Furthermore, there is a significant linear correlation between the three dimensions corresponding to entrepreneurship education in colleges and university students’ entrepreneurial self-efficacy based on the psychological elements. Based on the dimension of entrepreneurship innovation learning and the results of regression analysis, *R*^2^ = 0.397 and Adj.R^2^ = 0.392. The dimension of entrepreneurship innovation learning has a significant positive correlation with the entrepreneurial self-efficacy of college students. The specific performance is β = 0.551, *p* < 0.01, showing that H5 is reasonable. Based on the dimension of school organizational support, the regression analysis results show that *R*^2^ = 0.434 and Adj.R^2^ = 0.431. There is a significant positive correlation between the dimension of school organizational support and the entrepreneurial self-efficacy of college students. The specific performance is β = 0.623, *p* < 0.01. As a result, the rationality of H6 is verified. Based on the dimension of social interaction activities, the regression analysis results show that *R*^2^ = 0.464 and Adj.R^2^ = 0.457. The dimension of social interaction also has a significant positive correlation with the entrepreneurial self-efficacy of college students. The specific performance is β = 0.665, *p* < 0.01, showing that H7 is reasonable.

### Mediating Role of Entrepreneurial Self-Efficacy Under the Analysis of Psychological Factors

According to the hierarchical linear regression analysis method, the analysis results of the entrepreneurial self-efficacy under the psychological element analysis of college students in college entrepreneurship education and entrepreneurial performance are shown in Table 6.

**TABLE 6:** Test for the intermediary role of entrepreneurial self-efficacy in entrepreneurship education and entrepreneurial performance.

**Independent variable**	**Dependent variable**	**β**
Entrepreneurship and innovation learning	Entrepreneurial performance	0.257***
School organizational support		0.439***
Social interaction		0.238***
Intermediary variable		β_Iv_
Entrepreneurial self-efficacy		0.370***

*Symbol β indicates the beta coefficient. ^∗∗∗^p < 0.001.*

As shown in Table 6, according to the verification of the first two steps, when finally explaining the entrepreneurial performance of college students on the two levels of entrepreneurship education and college students’ entrepreneurial self-efficacy, it is found that for the dimension of entrepreneurship innovation learning, the corresponding β = 0.257, *p* < 0.001. For the dimension of school organizational support, the corresponding β = 0.439, *p* < 0.001. For the dimension of social interaction, the corresponding β = 0.238, *p* < 0.001. Therefore, entrepreneurial self-efficacy of college students has a good explanatory ability, i.e., entrepreneurial self-efficacy based on the analysis of psychological elements has a partial mediating effect. This shows that H8 is reasonable.

## Discussion

In the increasingly competitive network information society, the number of students and entrepreneurial teams developing entrepreneurship as a direction is also increasing (Huang-Saad and Celis, 2017). Therefore, college students were selected as the research samples in this study. The personality traits of entrepreneurial self-efficacy based on psychological element analysis were introduced. The correlations among college entrepreneurship education, entrepreneurial self-efficacy, and entrepreneurial performance were analyzed. The result of linear regression analysis of the correlation between entrepreneurship education in colleges and entrepreneurial performance of college students indicates that the school–enterprise alliance is an important aspect that can improve the level of entrepreneurial performance, which comes from the support of school-related organizations. Supporting the systematic learning of innovative entrepreneurship knowledge and technology that is beneficial to the entrepreneurial direction of college entrepreneurs will also have an impact on entrepreneurial performance. The impact of social interaction activities on entrepreneurial performance is obvious. College students and entrepreneurs can expand their interpersonal relationships by improving their communication skills and comprehensive abilities. With the help of social communication activities, they can develop more social relationships for start-ups, thereby promoting the improvement of corporate performance. After the three dimensions were compared corresponding to entrepreneurship education in colleges and universities, it was found that the support of school-related organizations has a greater impact on the performance of start-ups than entrepreneurship innovation learning and social interaction activities. With the support of the school-related organizations, the entrepreneurs of start-ups and college students can quickly carry out entrepreneurial activities. For the entrepreneurial group of college students, entrepreneurship education in colleges and universities has a crucial impact on them. The three dimensions of entrepreneurship innovation learning, school organizational support, and social interaction have a positive effect on the entrepreneurial performance of college students’ start-ups.

According to the previous analysis, the entrepreneurial self-efficacy of college students is a manifestation of college students’ psychological activities, which is a kind of personality traits and an expression of individual behavioral quality. It is analyzed based on psychological factors. The linear regression analysis results of the correlation between entrepreneurial self-efficacy and entrepreneurial performance of college students indicate that start-ups of college student entrepreneurs can utilize the entrepreneurial self-efficacy of students to improve their comprehensive ability, thereby affecting the entrepreneurship performance (Zhou and Wu, 2018; Zhu et al., 2019). Self-confidence helps individuals do things better, and the same is true for entrepreneurship. In this way, entrepreneurial performance can be substantially improved. Through a combination of the hierarchical linear regression analysis method with the BARON duality innovation intermediary three-part verification method, the intermediary role of entrepreneurial self-efficacy in college students’ entrepreneurship is verified. This is consistent with the conclusion that entrepreneurial self-efficacy has a moderating effect on entrepreneurial performance drawn by Miao et al. (2017). Through the linear regression statistical analysis of relevant data, the hypotheses were verified successively.

The linear regression analysis method is an important statistical analysis method. The correlation analysis of entrepreneurship education in colleges and universities, entrepreneurial self-efficacy, and entrepreneurial performance of college students proves this point with quantitative results. For the universality and scientificity of the research samples selected for the questionnaire survey, the reliability test and validity test of the scale tool are very important. Through the analysis of these two indicators, the validity of the scale tools was verified. Entrepreneurship performance is a key indicator to measure entrepreneurial ability and entrepreneurial level. At the same time, entrepreneurial psychology possessed by entrepreneurs has a direct or indirect influence on their entrepreneurial outcomes. Among college students, as a manifestation of their psychological activities, entrepreneurial self-efficacy also has an important impact on college entrepreneurship education and entrepreneurs combined with their personality traits. The research results based on linear regression analysis have also proved this point. Entrepreneurial self-efficacy is a component of mediating regulation. When analyzing entrepreneurship education based on psychological factors, it is very necessary to consider entrepreneurial self-efficacy.

## Conclusion

The concept of entrepreneurial self-efficacy based on the analysis of psychological factors was introduced. The research results of college entrepreneurship education and entrepreneurial performance show significant positive correlations among the three factors. Besides, the entrepreneurial self-efficacy serves as an intermediary role between entrepreneurship education and entrepreneurial performance. This fully proves the correlation between entrepreneurship education and entrepreneurial performance. This has a positive effect on analyzing the entrepreneurial behaviors of college students from a psychological perspective and promoting the improvement of entrepreneurial performance in the context of entrepreneurship education. However, there are still some limitations to the research process. Due to various factors, the research samples selected were merely universities in Xi’an, leading to a limited sample size. Besides, the selection of independent variables is not yet comprehensive and systematic. These aspects need to be strengthened in the future.

## Data Availability Statement

The raw data supporting the conclusions of this article will be made available by the authors, without undue reservation, to any qualified researcher.

## Ethics Statement

The studies involving human participants were reviewed and approved by the Xi’an Medical University Ethics Committee. The patients/participants provided their written informed consent to participate in this study.

## Author Contributions

KM: writing – original draft preparation. QS: resources and data curation. YM: validation and formal analysis. JT: writing – review and editing. All authors contributed to the article and approved the submitted version.

## Conflict of Interest

The authors declare that the research was conducted in the absence of any commercial or financial relationships that could be construed as a potential conflict of interest.
